# Silencing of TESTIN by dense biallelic promoter methylation is the most common molecular event in childhood acute lymphoblastic leukaemia

**DOI:** 10.1186/1476-4598-9-163

**Published:** 2010-06-24

**Authors:** Robert J Weeks, Ursula R Kees, Sarah Song, Ian M Morison

**Affiliations:** 1 Cancer Genetics Laboratory, Department of Biochemistry, University of Otago, PO Box 56, Dunedin 9054, New Zealand; 2 Telethon Institute for Child Health Research, University of Western Australia Centre for Child Health Research, Perth, Australia; 3 Department of Pathology, Dunedin School of Medicine, University of Otago, PO Box 913, Dunedin 9054, New Zealand

## Abstract

**Background:**

Aberrant promoter DNA methylation has been reported in childhood acute lymphoblastic leukaemia (ALL) and has the potential to contribute to its onset and outcome. However, few reports demonstrate consistent, prevalent and dense promoter methylation, associated with tumour-specific gene silencing. By screening candidate genes, we have detected frequent and dense methylation of the *TESTIN *(*TES*) promoter.

**Results:**

Bisulfite sequencing showed that 100% of the ALL samples (n = 20) were methylated at the *TES *promoter, whereas the matched remission (n = 5), normal bone marrow (n = 6) and normal PBL (n = 5) samples were unmethylated. Expression of *TES *in hyperdiploid, TEL-AML^+^, BCR-ABL^+^, and E2A-PBX^+ ^subtypes of B lineage ALL was markedly reduced compared to that in normal bone marrow progenitor cells and in B cells. In addition *TES *methylation and silencing was demonstrated in nine out of ten independent B ALL propagated as xenografts in NOD/SCID mice.

**Conclusion:**

In total, 93% of B ALL samples (93 of 100) demonstrated methylation with silencing or reduced expression of the *TES *gene. Thus, *TES *is the most frequently methylated and silenced gene yet reported in ALL. *TES*, a LIM domain-containing tumour suppressor gene and component of the focal adhesion complex, is involved in adhesion, motility, cell-to-cell interactions and cell signalling. Our data implicate *TES *methylation in ALL and provide additional evidence for the involvement of LIM domain proteins in leukaemogenesis.

## Background

Methylation of gene promoters is a mechanism by which tumour suppressor genes can be inactivated. The role of promoter methylation in carcinogenesis has been convincingly demonstrated when gene methylation constitutes one of two events causing inactivation of well-documented tumour suppressor genes. Examples include, familial stomach cancer in which the non-mutated allele of *CDH1 *is silenced by promoter methylation [1] and sporadic renal cell cancer and retinoblastoma in which the non-deleted alleles of *VHL *and *RB *respectively are silenced [2,3].

Distinction of genes whose methylation is causally associated with malignant transformation from those that are affected by non-specific methylation remains problematic. It is plausible that genes that are densely methylated in all cells within the leukaemic clone are more likely to be involved in tumourigenesis than those that are partially methylated in a low proportion of leukaemic cells. Also genes that are methylated in a high proportion of cases seem more likely to be pathogenically important. Parallel evidence of gene silencing and evidence that the affected gene is a tumour suppressor gene greatly strengthens the case for a causal role in tumourigenesis.

Promoter methylation can occur as a non-specific "bystander" event affecting genes that are already silent in non-malignant tissue. For example, Keshet *et al*. reported that of 106 genes whose promoters were methylated in colon cancer cell lines, 91 were already inactive in normal colon [4]. Similarly, in acute lymphoblastic leukaemia (ALL), the methylated gene *TIMP3 *was not expressed regardless of its methylation status [5].

Gene promoter methylation has been reported for an, as yet, small number of genes in ALL [5-9]. Genes that are reported to be methylated in ALL are involved in many cellular processes including growth regulation, apoptosis, cell adhesion, and others [6,8,9] and therefore gene silencing by methylation is hypothesised to be an important contributor to leukaemogenesis. An example of candidate epigenetic silencing in the initiation and progression of leukaemogenesis involves *CDKN2B*. This region frequently undergoes loss of heterozygosity (LOH) in ALL [10,11]. Dynamic changes of *CDKN2B *promoter methylation have been reported during human myeloid development [12] suggestive of a role in normal haematopoiesis. *CDKN2B *promoter methylation, detected by methylation specific PCR, has been repeatedly reported in ALL leading to claims that this methylation in involved in leukaemogenesis [13-16]. However, *CDKN2B *methylation was neither dense, clonal nor prevalent in the reported cases.

For most of the reported gene methylation events in ALL, the proportion of affected cells and the density of methylation have not been quantified. For example, positivity in a methylation specific PCR assay indicates the presence of methylated alleles, but not their relative proportions. Array based methods, although not quantitative, have also been used to screen for methylated genes [7].

Methylation-specific multiplex ligation-dependent probe amplification (MS-MLPA), a modification of MLPA, was developed as a tool for quantifying methylation at CpG sites located at methylation-sensitive restriction sites, by including a digestion step with a methylation-sensitive restriction enzyme such as HhaI [17]. MLPA can also be used to measure gene dose. Therefore, MS-MLPA allows the rapid, simultaneous analysis of both copy number and methylation at a number of gene promoters. Here we use MS-MLPA to quantify methylation of candidate tumour suppressor genes in paediatric ALL.

## Results

### TES methylation and expression in ALL bone marrow samples

To identify genes showing frequent high-level methylation, DNA from five ALL marrow samples and one peripheral blood sample were analysed. The density of methylation within 24 gene promoter regions was determined by using MS-MLPA, in multiple independent experiments. Percent methylation at the HhaI site was calculated by comparing normalised, HhaI-digested peak areas to normalised, mock-digested peak areas.

Of the genes examined, the *TES *promoter was the most frequently methylated, with four out of five ALL samples showing a high percentage of methylation. At the interrogated site, the percent methylation was 96, 76, 80, 75 and 7% for cases 1-5 respectively (normal PBL had 2.5% methylation).

**Figure 1:**
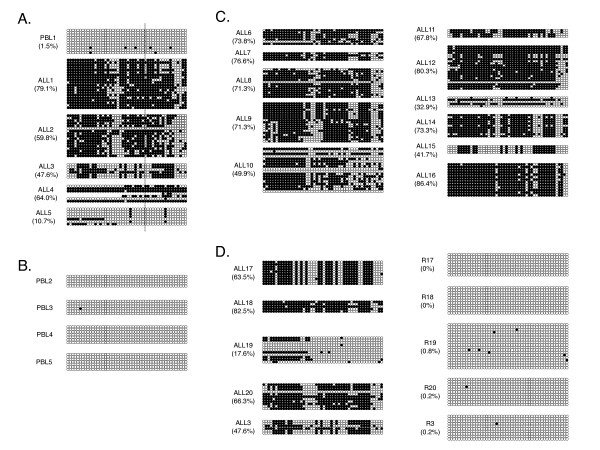
**Methylation plots for ALL bone marrow and normal PBL DNA**. Bisulfite sequencing of ALL marrow, remission marrow and normal PBL samples; each horizontal grouping indicates one sequenced clone, individual circles represent each CpG site (open circles and closed circles represent unmethylated and methylated CpG sites respectively). **A**. Methylation plots of ALL samples initially assayed by MS-MLPA. **B**. Normal PBL DNA methylation plots. **C**. Further ALL methylation plots. **D**. Matched ALL and remission methylation plots. Dotted line indicates location of HhaI site used in MS-MLPA. Percent methylation is shown in parentheses.

To validate the MS-MLPA results, detailed analysis of *TES *promoter (Additional file 1, Figure S1) methylation was obtained by performing bisulfite sequencing. ALL DNA samples and normal PBL DNA were bisulfite treated, amplified using bisulfite-specific primers, cloned and sequenced. Each of the ALL samples was hypermethylated compared to peripheral blood (Figure 1A). ALL5 (T lineage; Age: 7 years 7 months), for which methylation was not detected by MS-MLPA, showed the lowest level of methylation, but was methylated at CpG sites other than the HhaI site interrogated by MS-MLPA.

Bisulfite sequencing was then performed on a larger group of samples including normal peripheral blood, normal bone marrow, additional ALL bone marrow samples and remission bone marrow samples. Normal peripheral blood (n = 5; Figure 1B) and bone marrow (n = 6; data not shown) samples showed no methylation of the *TES *promoter. Leukaemia DNA, but not matched remission samples showed methylation of the *TES *promoter (Figures 1C and 1D). In summary, twenty out of twenty ALL samples were hypermethylated compared to normal blood and bone marrow at the *TES *promoter. Five of the ALL samples showed a small number of completely unmethylated alleles. These alleles could reflect the presence of a population of non-malignant cells in the marrow aspirate sample. For ALL samples, the percentage of methylation for all sites in the promoter ranged from 11% to 86% with a median of 65% and an interquartile range of 50-74%. In particular, B ALL samples had median methylation of 71% (interquartile range of 64-77%), whilst the T ALL samples (ALL5, ALL13 (6 years 1 month) and ALL15 (5 years 3 months)) had median methylation of 32.9% (B ALL vs. T ALL: p < 0.01 by T test). In contrast the percentage methylation with peripheral blood ranged from 0 to 1.5%. Since the amplification and cloning of bisulfite-treated DNA is subject to biases, the results are only semi-quantitative.

**Figure 2:**
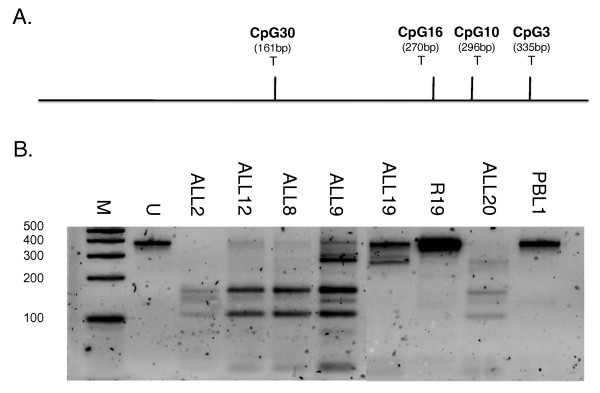
**CoBRA of ALL bone marrow DNA**. **A**. Diagram of reverse-strand bisulfite-specific PCR fragment showing location of potential Taq*^α^*I restriction sites (T). **B**. Taq*^α^*I digests of *TES *bisulfite-specific PCR products from normal PBL, ALL and remission samples. Fully methylated DNA will generate fragments of 161, 109, 26, 39 and 42 bp respectively. (Marker lane; 100, 200, 300, 400 and 500 bp bands; U - undigested PCR product).

To confirm that the observed hypermethylation did not reflect preferential cloning biases within the bisulfite conversion-derived PCR products, we developed a combined bisulfite restriction assay (CoBRA). After bisulfite conversion, reverse strand amplification of fully methylated DNA generates four Taq^α^I sites (TCGA) at CpG sites 3, 10, 16 and 30, whereas none are present in unmethylated DNA after amplification. CoBRA was performed on six ALL samples, one matched remission and one normal PBL sample. In all cases, the validity of the bisulfite sequencing results was confirmed by the CoBRA analysis (Figure 2). For example, ALL8 (B lineage; 5 years 9 months) and ALL12 (B lineage; 5 years), which show dense methylation by bisulfite sequencing (Figure 1C), showed a CoBRA banding pattern indicative of dense methylation. Similarly, the partial methylation shown by ALL19 (B lineage; 6 years 3 months) (Figure 1D) is paralleled by a CoBRA banding pattern indicative of partial methylation at CpG10 and CpG16. The unmethylated remission and normal PBL were undigested by Taq^α^I, also in agreement with bisulfite sequencing results.

The presence of SNPs (rs1319886, rs28411392 and rs11549785) in the *TES *promoter permitted analysis of allele-specific methylation. Bisulfite sequencing of the reverse strand confirmed biallelic methylation in five informative ALL samples (Table 1). Additionally the presence of another promoter SNP (rs11549786), outside of the bisulphite PCR amplicon, and of a telomeric microsatellite (D7s655) permitted copy number analysis. In 15 out of 20 cases LOH was excluded, but in the remaining 5 non-informative cases LOH could not be excluded.

**Table 1:** Summary of leukaemia methylation and LOH status in ALL bone marrow aspirates

	Bisulfite-specific data		Genomic DNA			
	Bisulfite (rev) SNP^a^	Biallelic Methylation	D7s655^b^	Genotype SNP^c^	LOH^d^	Notes
**ALL1**			2	C/T, C/T, C/T, A	N	Same as remission

**ALL2**	GAG		2	C, T, C, A	N	

**ALL3**	GAG, AAG	Y	1	C, T, C/T, A	N	Same as remission

**ALL4**			1	C/T, C/T, C/T, A	N	

**ALL5**			1	C, T, C/T, A	N	

**ALL6**			2		N	

**ALL7**			2		N	

**ALL8**	GAG, AAG	Y	1		N	

**ALL9**	GGA, AAG	Y			N	

**ALL10**	GAG, GGA	Y			N	

**ALL11**			2		N	

**ALL12**	GAG		2		N	

**ALL13**						LOH?

**ALL14**			2		N	

**ALL15**			1		Non-Informative	

**ALL16**	AAG					LOH?

**ALL17**					Non-Informative	LOH?

**ALL18**	GGA, GAG	Y	1	C/T, C/T, C, A	N	Same as remission

**ALL19**	GGA				Non-Informative	Same as remission

**ALL20**	GGA		1		Non-Informative	LOH?

^a ^SNP data (rs1319886, rs2811392, rs11549785) generated from bisulfite sequencing. ^b^Number of alleles at D7s655 microsatellite. ^c^SNP data (rs11549786, rs11549785, rs2811392, rs1319886) generated from promoter sequencing. ^d ^Summary of loss of heterozygosity status.

*TES *mutations have been reported in T ALL (CCRF-CEM), breast cancer and ovarian cancer cell lines [18,19] and we hypothesised that the less methylated ALL samples may contain coding mutations. Exon sequences from ALL5 and ALL19 (the two samples with the lowest density of methylation) were amplified and sequenced. No mutations were found (data not shown), however we were unable to exclude the possibility of intragenic deletions not readily detectable by amplification and sequencing.

*TES *promoter methylation has been shown to down-regulate expression in breast cancer cell lines, leukaemia cell lines [18] and glioblastomas [20]. To determine the role of methylation on *TES *expression in ALL we used microarrays to compare expression in a separate cohort of paediatric precursor B ALL specimens with two control precursor cell populations, normal bone marrow CD34+ progenitor cells (n = 5) and umbilical cord blood CD19+IgM- (pre-B) cells (n = 3). Leukaemia samples were classified into subtypes using expression profiling based on Yeoh *et al*. [21].

**Figure 3:**
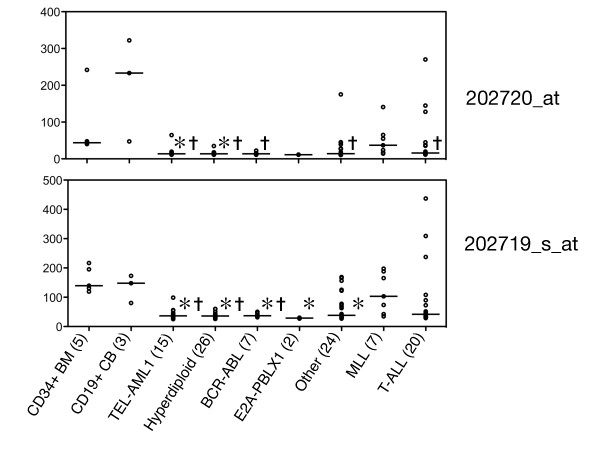
***TES *expression levels in ALL subgroups**. *TES *expression in 102 independent ALL samples was assayed using Affymetrix HG-U133A microarray. *TES *expression was calculated for both *TES*-specific probes (probe 202719_s_at (lower) is located in *TES *coding sequence and probe 202720_s_at (upper) is located in the 3' UTR). A horizontal line indicates the median TES expression for each sub-group. (* Indicates significance (p < 0.01) to CD34+ median expression; ✝ indicates significance (p < 0.01) to CD19+ median expression)

As previously reported, in a comparison of gene expression between normal bone marrow CD34+ cells and precursor B ALL, *TES *was among the most down-regulated genes as assessed by Affymetrix HG-U133A microarrays [22]. Under a linear regression model a marked reduction in relative expression of *TES *(probe-set 202719_s_at) was observed in the common subtypes of precursor B ALL, including hyperdiploid, TEL-AML1, BCR-ABL, E2A-PBX1 (for each subgroup p < 0.001) and in T lineage ALL (p < 0.05) but not in MLL rearranged ALL (p = 0.20), when compared to CD34+ BM (Figure 3). Similar p-values were recorded when CD19+ CB was used as the comparison or if the second *TES *probe-set (202120_at) was used, though p-values were not always as strong. In total marked reduction in *TES *expression was observed in 68 of the 74 cases of B ALL (Figure 3).

### TES methylation and expression in leukaemia cell lines

**Figure 4:**
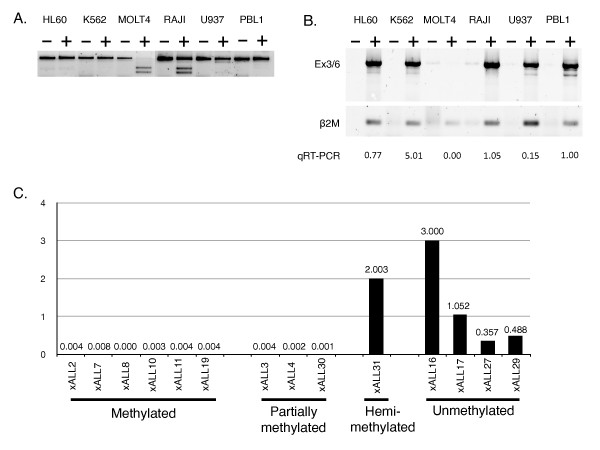
***TES *methylation and expression in cell lines and xenografts**. **A**. Taq*^α^*I digests of *TES *bisulfite-specific PCR products from cell lines HL60, K562, MOLT4, Raji and U937, and normal PBL. **B**. Amplification of cell lines and normal PBL samples cDNAs using primers specific for *TES *exon 3 (^5' ^TGCAAGTGTGGCCAAGAAGAGC ^3'^) and exon 6 (^5' ^CAGCGTGATTCTTCACATAGCAGG ^3'^) and *β2-microglobulin *(For: ^5' ^GAGTATGCCTGCCGTGTG ^3' ^and Rev: ^5' ^AATCCAAATGCGGCATCT ^3'^); first strand cDNA, with (+) or without (-) Superscript III reverse transcriptase. *TES *expression levels relative to PBL expression after normalisation to *β2-microglobulin *were calculated using qRT-PCR and are shown. **C**. qRT-PCR was used to calculate *TES *expression levels in ALL xenografts relative to PBL expression after normalisation to *β2-microglobulin *expression. Methylation status was determined using CoBRA.

The relationship between *TES *expression and methylation was examined directly in available leukaemia cell lines. DNA methylation status was determined using CoBRA as before (Figure 4A). In brief, MOLT4 (T ALL) bisulfite-specific PCR product was completely digested by Taq^α^I, indicating that MOLT4 cells are methylated at the four CpG sites interrogated by Taq^α^I. Raji (Burkitt lymphoma) cells appear to be hemi-methylated, *i.e.*, methylated on one allele and unmethylated on the other allele. RT-PCR was performed on total cell line RNA using exon-specific primer pairs. PCR products from amplifications with primers spanning exon 3 and exon 6 are shown (Figure 4B). *TES *expression was present in Raji and normal PBL, but was undetectable in MOLT 4 cells by RT-PCR. Also, *TES *expression levels were quantified using real-time quantitative RT-PCR (qRT-PCR) with expression levels calculated relative to normal PBL levels after normalisation to *β2-microglobulin *and reproduced on Figure 4B. *TES *promoter methylation resulted in a reduction of *TES *expression in the leukaemia cell lines tested in agreement with previously published reports [18,20].

To confirm the inverse relationship between methylation and expression, CoBRA and qRT-PCR was performed on *TES *expressing, myeloid-lineage cell lines: HL60, K562 and U937. Bisulfite-specific PCR products from HL60, K562 and U937 cells were not digested by Taq^α^I, thus demonstrating the inverse relationship between methylation and expression.

As previously reported [18] multiple splice variants of *TES *were detectable by RT-PCR; however an unexpected splice variant was amplified from both cell lines and normal blood (Figure 4B). This novel splice variant was sequenced and compared with published variants. The novel variant cDNA uses a cryptic splice site present in exon 5 to generate a 102 bp shorter cDNA product, which if translated would result in a truncated protein of 237 amino acids (see Additional file 2, Figure S2).

### TES silencing in ALL xenografts

Next, fourteen ALL xenografts [23] were analysed for *TES *promoter methylation and expression analysis. Methylation status was measured by CoBRA assay and was in complete, reciprocal agreement with expression levels measured by real time RT-PCR (see Figure 4C). Without knowledge of their phenotypes, we predicted that almost all methylated and non-expressing xenografts would be B-lineage derived, whereas unmethylated and *TES *expressing xenografts would be T-lineage ALL. xALL 2, 3, 4, 7, 8, 10, 11, 19 and 30 were predicted to be B-lineage: this was correct except for xALL 30 which was T ALL. xALL 16, 17, 27, 29 and 31 were predicted to be T ALL: xALL 16, 27, 29 and 31 were confirmed to be T-lineage ALL, whereas xALL 17 was B-lineage [23]. Analysis of the karyotype of xALL 17 (46, XX, -20, +21 (18), XX, -20, +21, +mar (4)) suggests that xALL 17 be classified as "Other" according to Yeoh *et al*. [21] (see Figure 3). Of the *TES-*expressing xenografts, xALL31 appeared to show hemi-methylation of the *TES *promoter with 50% of the bisulfite-specific PCR product not being digested by Taq^α^I. This hemi-methylation was similar to that seen with the Raji cell line (see Figure 4A). In conclusion, we were able to predict the lineage of ALL xenografts on the basis of *TES *expression and methylation.

We have demonstrated *TES *hypermethylation in 17 of 17 B ALL samples using bisulfite sequencing and down-regulation of *TES *expression using expression profiling in 68 of 74 independent B ALL samples. In addition, we have demonstrated both promoter methylation and silencing of *TES *expression in 8 of 9 B ALL xenografts. In total, transcriptional silencing and/or promoter methylation of *TES *occurred in 93 of 100 B ALL and 20 of 28 T ALL cases.

## Discussion

This study demonstrates that the *TES *promoter is densely methylated in a high proportion of childhood ALL. Eighteen of twenty tested leukaemia bone marrow aspirate samples were densely methylated at the *TES *promoter, whereas matched remission marrow, normal peripheral blood and bone marrow samples were unmethylated. In addition, eight of nine B-lineage ALL xenograft samples showed dense methylation of the *TES *promoter. The proportion of ALL cases that showed methylation of *TES *is among the highest for any genes reported to date [6] and similar to levels reported by Hesson *et al*. [24] and Taylor *et al*. [7]. Taylor *et al. *found methylation in *DCC *in 9 of 10 cases of precursor B ALL and in *RUNDC3B*, *KCNK2*, and *DLC1 *in 7, 7 and 8 (respectively) of these 10 cases [7]. And in a recent study, Hesson *et al*. reported *RASSF6 *promoter methylation in 48 of 51 B ALL and 12 of 29 T ALL cases [24]. For comparison, some of the genes that are commonly referred to as methylated in ALL, *e.g*. the *p15 *promoter have shown methylation of only 18% of alleles [6].

*TES *is located in 7q31.2, a region showing frequent loss of heterozygosity in myeloid malignancies [25] (between D7S2554 and D7S2460). In addition, loss of heterozygosity at 7q31 occurs in gastric cancer [26], prostate cancer [27], breast cancer [28] and others (see Tobias *et al*. for an overview [19]). The frequent LOH implies the presence of at least one tumour suppressor gene, although the absence of mutations in candidate genes has led to suggestions that regulatory gene(s) within the region might be inactivated by epigenetic mechanisms [29,30]

*TES *is a putative tumour suppressor gene. Drusco *et al*. concluded that *TES *acted as a tumour suppressor gene *in vivo*, given that *TES *knockout mice showed an increased susceptibility to carcinogen (nitrosomethylbenzylamine) - induced gastric cancer [31]. In addition, restoration of *TES *by adenoviral transduction of non-*TES *expressing breast cancer and uterine sarcoma cell lines inhibited their growth by induction of apoptosis [32]. Additionally the tumourigenic potential of these transduced cell lines was significantly reduced in nude mice. Furthermore, forced *TES *expression in a non-expressing, invasive ductal breast carcinoma cell line had an inhibitory effect on proliferation, on anchorage-independent growth in agarose and on colony forming ability [33].

Available evidence suggests that apart from deletion, the commonest mechanism of *TES *inactivation is epigenetic. *TES *methylation has been shown in primary tumours, including glioblastomas (18 of 31) [20] and ovarian cancer [19]. Methylation at a single site in the *TES *promoter has been reported for several cell lines including lymphoid leukaemia, breast cancer and pancreatic cancer cells [18]. *TES *methylation is closely associated with loss of *TES *expression in cell lines [18] and in glioblastoma cells [20]. By using xenograft and immortalised leukaemia cell lines, we have now directly demonstrated the reciprocal relationship between methylation and expression.

In contrast to epigenetic inactivation, *TES *mutations have been reported in only three cell lines [18,19]. We investigated whether ALL5 and ALL19 (both partially methylated) harboured mutations; however *TES *coding mutations were not detected by exon sequencing.

We confirmed that there is substantial down-regulation of *TES *expression in an independent cohort of ALL cases and in B ALL xenografts. The marked down-regulation of *TES *in virtually all cases of ALL, compared to normal precursor cells, indicates that *TES *methylation suppresses expression that is present in relevant precursor cells. Although *TES *expression levels were not confirmed in these ALL cases, we had previously demonstrated excellent correlation between these array results and qRT-PCR measurements for the majority (33/48) of selected genes [34]. Additionally, our array results show consistent down-regulation with both *TES *- specific probes used. Importantly another published series of 87 cases of B-lineage ALL showed that *TES *was downregulated compared to normal bone marrow and normal haematopoietic cells, being the second most highly ranked down-regulated gene [35]. Furthermore, within the cohort of Ross *et al*., *TES *showed substantial down-regulation in B-lineage ALL compared to MLL-translocation ALL, or T-cell ALL [36].

TES, a highly conserved protein (Additional file 3, Figure S3), is composed of three C-terminal LIM domains and a PET (prickle, espinas and testin) domain of unknown function. LIM domains are 50-60 amino acids in size and are believed to be involved in protein-protein interactions. LIM-domain containing proteins are classified into 4 groups; groups 2, 3 and 4 being predominantly localised to cytoskeleton-associated structures including focal adhesion complexes, whereas group 1 LIM proteins are predominantly nuclear.

TES, a group 3 LIM domain protein, is a component of the focal adhesion complex and localises to cell-matrix adhesions, cell-cell contacts and to actin stress fibres. In mice, Tes has been shown to interact or colocalise with cytoskeletal proteins including actin, zyxin, Mena, VASP, talin, α-actinin, and paxillin [37]. Tes recruitment to the focal adhesion complex appears to be mediated by zyxin through the LIM1 domain [33]. Tes also binds Mena, which inhibits Mena's ability to interact with FPPPP-motif proteins, such as zyxin, thus displacing Mena from focal adhesion complexes [38]. Over-expression of TES leads to loss of Mena from focal adhesions, increased cell spreading and decreased cell motility [37-40]. This suggests that Tes downregulates Mena-dependent cell motility, implying that loss of Tes might enhance cell mobility [38]. As haematopoietic development requires coordinated bone marrow retention, adhesion and cell migration [41], we suggest that silencing of *TES *might contribute to ALL by interfering with normal interactions and adhesion between progenitors and stroma, with increased motility of immature progenitors, resulting in premature release of progenitors from bone marrow niches.

Additionally, TES has been detected in the nucleus, specifically the nucleolus, and the endoplasmic reticulum [42]. It is proposed that the nucleolar localisation involves an alternative, closed confirmation state in which the N-terminus binds to the third LIM domain of TES [42]. Therefore TES, similar to many of the other cytoplasmic LIM proteins, may shuttle into, and have a functional role within the nucleus. The possibility of additional complexity in the functional roles of TES is raised by our identification of a previously unreported short transcript of *TES *in normal cells. If translated, this truncated protein would be very similar to the LIM-less TES proteins designed by Coutts *et al*. [37]. These LIM-less TES proteins do not localise to focal adhesions but are found associated with actin stress fibres. The function of the LIM-less splice variant is unknown, but it may compete with full-length TES activity.

Interestingly, other LIM domain proteins have been implicated in leukaemogenesis. For example, the group 1 nuclear-localised LIM protein LMO2 acts as an oncogenic protein in T-cell ALL [43]. The role of LMO2 in oncogenesis was also demonstrated in two separate gene therapy trials for X-linked SCID, which were halted when five (out of 19) patients developed leukaemia. Remarkably four of the patients had insertion of the therapeutic retrovirus upstream of the LMO2 locus with subsequent over-expression of the LMO2 gene [44]. LMO2 appears to act as a bridging molecule to assemble haematopoietic transactivating complexes and is essential for development of haematopoietic lineages [45].

## Conclusions

The current study shows that dense methylation of the *TES *promoter, resulting in loss of *TES *expression is prevalent in childhood ALL. Indeed, *TES *has been shown to be either methylated or silenced in 93% of B ALL (93 of 100) tested; thus *TES *is the most common (epi)genetic abnormality in B ALL. In addition a recent study reports *TES *as one of 36 genes showing hypermethylation and reduced expression in *ETV6/RUNX1*-positive and hyperdiploid subtypes of B ALL [9]. Based on the known functions of TES, we predict consequent aberrant adhesion between progenitors and bone marrow stroma, which we hypothesise contributes to leukaemia initiation and progression. Evidence that TES, like other LIM proteins, is shuttled to the nucleus and the presence of additional transcripts of *TES*, invoke the possibility that the tumour suppressing functions of TES may reside within alternative functions that are yet to be determined.

## Methods

### ALL samples

Paediatric acute lymphoblastic leukaemia (n = 20) and matched remission (n = 5) bone marrow aspirate samples, normal marrow and normal peripheral blood leukocyte (PBL) samples were collected in accord with ethical approval obtained from the Otago Ethics Committee. All samples contained at least 80% blasts, and usually at least 90%. The remission samples that were used were obtained at least 35 days after diagnosis. The median age of the ALL patients was 4 years 10 months (range 2 years to 11 years 3 months), and comprised 17 B lineage and 3 T lineage (ALL5, ALL13 and ALL15) ALL. Samples were obtained prior to routine molecular testing; two B lineage cases showed a hyperdiploid karyotype (ALL11 and ALL14); one B lineage case (ALL2) had t(9;22) and no cases showed structural 11q23 abnormalities (MLL gene location).

Cell lines were newly obtained from the American Type Culture Collection (ATCC, Manassas, VA, USA) and maintained according to ATCC instructions.

A separate group of ALL samples were maintained as xenografts in SCID/NOD mice as previously described [23].

### Multiplex Ligation-dependent Probe Amplification assay

Twenty-four candidate genes were chosen from published literature. At the time of selection (2005) these candidates included the majority of genes known to be methylated in haematopoietic cancers.

MS-MLPA probes were designed to interrogate HhaI sites within the reported region of methylation and purchased from Sigma-Proligo (St Louis, MO, USA)(see Additional file 4, Table S4). The 24 HhaI site containing probes and three control probes were prepared as three separate probe-mixes and used with the MLPA EK1 kit (MRC Holland, Amsterdam, The Netherlands). MS-MLPA assays were performed as per manufacturer's instructions. After probe ligation each sample was divided in two, with one aliquot being incubated with HhaI. Ligated probes were then amplified using MLPA-universal primers and products were detected with an ABI3100 capillary sequencer, and analysed using GENESCAN (Applied Biosystems Ltd, Foster City, CA, USA) and EXCEL (Microsoft, Redmond, WA, USA) software. Three control probes, not digested by HhaI, were used for normalisation of probe peak areas.

Each probe peak area was normalised using the sum of the control probe peak areas. Percent methylation was calculated from the normalised HhaI-digested peak area and the normalised mock-digested peak area (as below).

### Bisulfite Sequencing

DNA from ALL and matched remission samples, normal PBL and bone marrow samples were bisulfite-treated using either the EZ DNA Methylation Gold system (Zymo Research, Orange, CA, USA) or our standard bisulfite-treatment protocol [46].

Bisulfite-specific primers were designed using the MethPrimer website [47] (Forward: ^5' ^TTAGGGTTATTGAGTTTGTTTAGTAGG ^3'^; Reverse: ^5' ^CTTTATTTTCCAAATCCATATTAAC ^3'^), and were used to amplify 371 base pairs or 48 CpG dinucleotides of the *TES *promoter. Amplified products were cloned using TOPO TA Cloning system (Invitrogen, Carlsbad, CA, USA) and sequenced using ABI BigDye 3.1 Terminator System and supplied TOPO M13 reverse primer. Resulting sequences were viewed and, if necessary, edited in 4 Peaks http://mekentosj.com/4peaks/, and aligned using the Se-Al v2.0a11 Carbon program http://evolve.zoo.ox.ac.uk/. Percent methylation was calculated as the percentage of methylated CpGs out of total CpGs sequenced.

### Genotyping

Amplification of genomic DNA was performed using *TES *promoter-specific primers (Forward: ^5' ^ACCAGGTCAGGGTCACTGAGCTTGC ^3'^; Reverse: ^5' ^ACCCGCGCAGGTGAAGCAGC ^3'^) to investigate four SNPs (rs11549786, rs11549785, rs28411392 and rs1319886). PCR products were purified using DNA Clean-Up kit (Zymo Research) and sequencing was performed as above, using genotyping PCR primers.

### Combined Bisulfite Restriction Assay (CoBRA)

CoBRA was designed to interrogate four Taq^α^I sites generated from methylated DNA after bisulfite conversion. DNA was bisulfite-treated and amplified using primers designed to the reverse strand (Forward: ^5'^ATTTTGTTTTTTAGGTTTATGTTAA ^3'^; Reverse: ^5' ^CCAAATCAAAATCACTAAACTTACC ^3'^). After PCR amplification and DNA Clean-Up (Zymo Research), purified products were digested with Taq^α^I and electrophoresed through 2% Seakem LE agarose (Lonza, Basel, Switzerland). Amplified products were cloned and sequenced as above, to generate SNP genotyping data.

### Microarray Gene expression

Total RNA was extracted from 101 bone marrow specimens from paediatric ALL patients, from five isolates of CD34+ cells from normal marrow, and from three isolates of CD19+IgM- cells from umbilical cord blood as previously described [22]. cRNA was prepared and hybridised to HG-U133A microarray (Affymetrix, Santa Clara, CA, USA) as described [22]. ALL samples were classified into leukaemia sub-groups, as described by Yeoh *et al*. [21]. *TES *expression was calculated from both *TES*-specific probes and median expression level for each ALL sub-group was calculated.

### Reverse Transcriptase PCR

First strand cDNA was generated from total RNA using Superscript III Reverse Transcriptase (Invitrogen) and manufacturer's instructions, except a mix of oligo-dT and random primers were used to prime the synthesis. Multiple exon-specific primers were designed to amplify cDNA, with genomic DNA not being amplified or producing larger sized products. RT-PCR products were cloned using the TOPO TA Cloning System before being sequenced. *TES *expression levels were measured using ABI Assay-On-Demand kits (*TES*: Hs_00932509_g1; *β2-microglobulin*: Hs00984230_m1) and ABI PRISM 7900 HT Sequence Detection System (Applied Biosystems Ltd) according to manufacturer's instructions. *TES *expression levels were normalised to *β2-microglobulin *before calculation relative to normal PBL levels.

## Competing interests

The authors declare that they have no competing interests.

## Authors' contributions

RJW and IMM designed the research and co-wrote the manuscript. RJW performed all the experiments, except for the microarray expression experiments (URK), and RJW, URK, SS and IMM performed data analysis. All authors read and approved the final manuscript.

## Supplementary Material

Additional file 1 **Figure S1: *TES *promoter CpG island sequence**. *TES *promoter CpG island sequence showing the location of 48 CpG sites (numbered), the translation start site (arrow) and three SNPs (rs1319886, rs2811392 and rs11549785). MS-MLPA interrogated HhaI restriction site is underlined. Click here for file

Additional file 2 **Figure S2: Splice variant sequence**. Alignment of *TES *cDNA region (exon 3 to exon 6) (GenBank ID AK222840; upper) with PCR-generated splice variant (GQ423971; lower); the missing 102 bp fragment and predicted, truncated protein sequences are shown. Primer sequences are shown underlined and exon splice sites are indicated by a hyphen. Click here for file

Additional file 3 **Figure S3: Alignment of TES proteins**. Human TES proteins aligned with TES proteins of other species. PET and LIM domains are indicated. Click here for file

Additional file 4 **Table S4: MS-MLPA probe sequences**. Click here for file
