# Supplementary material for: Silencing of TESTIN by dense biallelic promoter methylation is the most common molecular event in childhood acute lymphoblastic leukaemia
Source: Mol Cancer. 2010 Jun 24;9:163. doi: 10.1186/1476-4598-9-163 (PMC3224738; doi:10.1186/1476-4598-9-163)
Supplement: Additional file 1 — Figure S1: TES promoter CpG island sequence. TES promoter CpG island sequence showing the location of 48 CpG sites (numbered), the translation start site (arrow) and three SNPs (rs1319886, rs2811392 and rs11549785). MS-MLPA interrogated HhaI restriction site is underlined. [file 1476-4598-9-163-S1.PDF]

TCAGGGTCACTGAGCTTGCCCAGCAGGG**CG**[1]C**CG**[2]CCT**CG**[3]GA**CG**[4]C**CG**[5]CCC**CG**[6]CCT[a]CTCACCTT

GCCAG**CG**[7]C**CG**[8]**CG**[9]CT**CG**[10]GGC**CG**[11]**CG**[12]AAGGTG**CG**[13]TG**CG**[14]G**CG**[15]CT**CG**[16]GTGATTGG**CG**[17]G

CG[18]GCC**CG**[19]GAGCTGCC**CG**[20]GCTGCCATTGGCTGCC**CG**[21]GCCCC[b]CTTTGTTCC**CG**[22]GGTC**CG**[23]

GGC**CG**[24]CAGGCC**CG**[25]CTG**CG**[26]G**CG**[27]GACTGGG**CG**[28]G**CG**[29]GAAGTT**CG**[30]A**CG**[31]G**CG**[32]C**CG**[33]GG

CG[34]AGTGGCTGTTGAG**CG**[35]G**CG**[36]C**CG**[37]**CG**[38]GGAGTTC**CG**[39]CAGGTTTCC[c]**CG**[40]TGTT**CG**[41]CAG

CG[42]GAGC**CG**[43]GAGGCCAGCTGAACC**CG**[44]GC**CG**[45]TGGGATCC**CG**[46]GATAGGAGGAGGGGACC

CATAGGA**CG**[47]**CG**[48]TTAACATGGACCTGGAAAACAAAG

[a] = rs1319886 (A/G)
[b] = rs28411392 (C/T)
[c] = rs11549785 (C/T)
