# Supplementary material for: Silencing of TESTIN by dense biallelic promoter methylation is the most common molecular event in childhood acute lymphoblastic leukaemia
Source: Mol Cancer. 2010 Jun 24;9:163. doi: 10.1186/1476-4598-9-163 (PMC3224738; doi:10.1186/1476-4598-9-163)
Supplement: Additional file 2 — Figure S2: Splice variant sequence. Alignment of TES cDNA region (exon 3 to exon 6) (GenBank ID AK222840; upper) with PCR-generated splice variant (GQ423971; lower); the missing 102 bp fragment and predicted, truncated protein sequences are shown. Primer sequences are shown underlined and exon splice sites are indicated by a hyphen. [file 1476-4598-9-163-S2.PPT]

## Slide 1
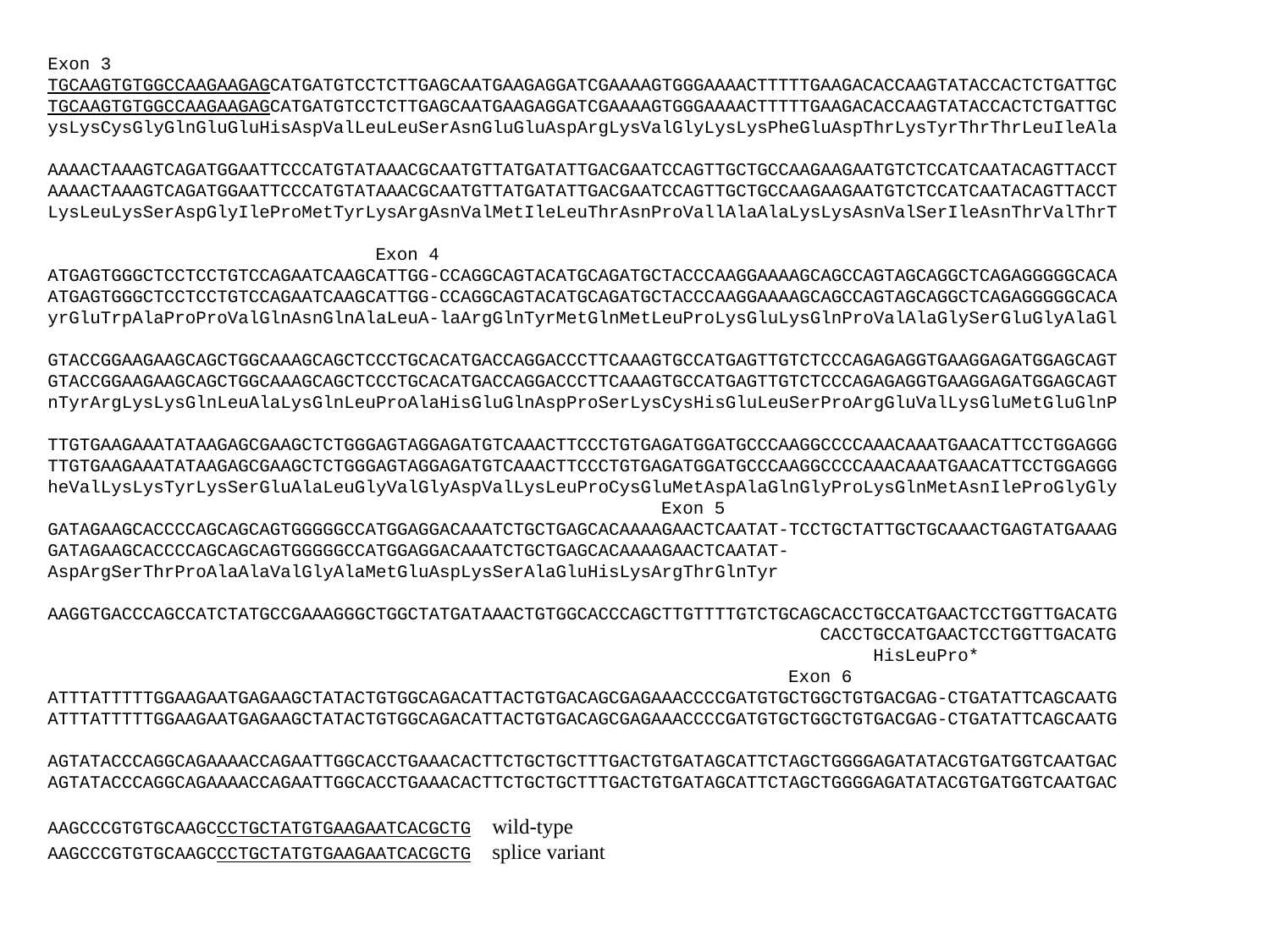

Exon 3
TGCAAGTGTGGCCAAGAAGAGCATGATGTCCTCTTGAGCAATGAAGAGGATCGAAAAGTGGGAAAACTTTTTGAAGACACCAAGTATACCACTCTGATTGC
TGCAAGTGTGGCCAAGAAGAGCATGATGTCCTCTTGAGCAATGAAGAGGATCGAAAAGTGGGAAAACTTTTTGAAGACACCAAGTATACCACTCTGATTGC
ysLysCysGlyGlnGluGluHisAspValLeuLeuSerAsnGluGluAspArgLysValGlyLysLysPheGluAspThrLysTyrThrThrLeuIleAla
AAAACTAAAGTCAGATGGAATTCCCATGTATAAACGCAATGTTATGATATTGACGAATCCAGTTGCTGCCAAGAAGAATGTCTCCATCAATACAGTTACCT
AAAACTAAAGTCAGATGGAATTCCCATGTATAAACGCAATGTTATGATATTGACGAATCCAGTTGCTGCCAAGAAGAATGTCTCCATCAATACAGTTACCT
LysLeuLysSerAspGlyIleProMetTyrLysArgAsnValMetIleLeuThrAsnProVallAlaAlaLysLysAsnValSerIleAsnThrValThrT
					 Exon 4
ATGAGTGGGCTCCTCCTGTCCAGAATCAAGCATTGG-CCAGGCAGTACATGCAGATGCTACCCAAGGAAAAGCAGCCAGTAGCAGGCTCAGAGGGGGCACA
ATGAGTGGGCTCCTCCTGTCCAGAATCAAGCATTGG-CCAGGCAGTACATGCAGATGCTACCCAAGGAAAAGCAGCCAGTAGCAGGCTCAGAGGGGGCACA
yrGluTrpAlaProProValGlnAsnGlnAlaLeuA-laArgGlnTyrMetGlnMetLeuProLysGluLysGlnProValAlaGlySerGluGlyAlaGl
GTACCGGAAGAAGCAGCTGGCAAAGCAGCTCCCTGCACATGACCAGGACCCTTCAAAGTGCCATGAGTTGTCTCCCAGAGAGGTGAAGGAGATGGAGCAGT
GTACCGGAAGAAGCAGCTGGCAAAGCAGCTCCCTGCACATGACCAGGACCCTTCAAAGTGCCATGAGTTGTCTCCCAGAGAGGTGAAGGAGATGGAGCAGT
nTyrArgLysLysGlnLeuAlaLysGlnLeuProAlaHisGluGlnAspProSerLysCysHisGluLeuSerProArgGluValLysGluMetGluGlnP
TTGTGAAGAAATATAAGAGCGAAGCTCTGGGAGTAGGAGATGTCAAACTTCCCTGTGAGATGGATGCCCAAGGCCCCAAACAAATGAACATTCCTGGAGGG
TTGTGAAGAAATATAAGAGCGAAGCTCTGGGAGTAGGAGATGTCAAACTTCCCTGTGAGATGGATGCCCAAGGCCCCAAACAAATGAACATTCCTGGAGGG
heValLysLysTyrLysSerGluAlaLeuGlyValGlyAspValLysLeuProCysGluMetAspAlaGlnGlyProLysGlnMetAsnIleProGlyGly
									 Exon 5
GATAGAAGCACCCCAGCAGCAGTGGGGGCCATGGAGGACAAATCTGCTGAGCACAAAAGAACTCAATAT-TCCTGCTATTGCTGCAAACTGAGTATGAAAG
GATAGAAGCACCCCAGCAGCAGTGGGGGCCATGGAGGACAAATCTGCTGAGCACAAAAGAACTCAATAT-
AspArgSerThrProAlaAlaValGlyAlaMetGluAspLysSerAlaGluHisLysArgThrGlnTyr
AAGGTGACCCAGCCATCTATGCCGAAAGGGCTGGCTATGATAAACTGTGGCACCCAGCTTGTTTTGTCTGCAGCACCTGCCATGAACTCCTGGTTGACATG
 CACCTGCCATGAACTCCTGGTTGACATG
									 			HisLeuPro*
											 Exon 6
ATTTATTTTTGGAAGAATGAGAAGCTATACTGTGGCAGACATTACTGTGACAGCGAGAAACCCCGATGTGCTGGCTGTGACGAG-CTGATATTCAGCAATG
ATTTATTTTTGGAAGAATGAGAAGCTATACTGTGGCAGACATTACTGTGACAGCGAGAAACCCCGATGTGCTGGCTGTGACGAG-CTGATATTCAGCAATG
AGTATACCCAGGCAGAAAACCAGAATTGGCACCTGAAACACTTCTGCTGCTTTGACTGTGATAGCATTCTAGCTGGGGAGATATACGTGATGGTCAATGAC
AGTATACCCAGGCAGAAAACCAGAATTGGCACCTGAAACACTTCTGCTGCTTTGACTGTGATAGCATTCTAGCTGGGGAGATATACGTGATGGTCAATGAC
AAGCCCGTGTGCAAGCCCTGCTATGTGAAGAATCACGCTG	wild-type
AAGCCCGTGTGCAAGCCCTGCTATGTGAAGAATCACGCTG	splice variant
