# Supplementary material for: Silencing of TESTIN by dense biallelic promoter methylation is the most common molecular event in childhood acute lymphoblastic leukaemia
Source: Mol Cancer. 2010 Jun 24;9:163. doi: 10.1186/1476-4598-9-163 (PMC3224738; doi:10.1186/1476-4598-9-163)
Supplement: Additional file 3 — Figure S3: Alignment of TES proteins. Human TES proteins aligned with TES proteins of other species. PET and LIM domains are indicated. [file 1476-4598-9-163-S3.DOC]

Homo 1 MDLENKVKKMGLGHEQGFGAPCLKCKEKCEGFELHFWRKICRNCKCGQEEHDVLLSNEEDRKVGKLFEDTKYTTLIAKLK

Homo 2 -----------------------------------------------------------------------

Chimp --------------------------------------------------------------------------------

Gorilla -----------------------------------------------------------------------

Orangutan --------------------------------------------------------------------------------

Sheep ----A---------------------------------------------------------------------------

Cattle ----A---------------------------------------------------------------------------

Mouse 1 ------------------N------------------------------------R---------------

Mouse 2 ----T-M--------------------N------------------------------------R---------------

Rat ----T-M--------------------N----------------------------T-------R---------------

Xenopus -E----F--VT----E-S---------------------------------S--SN--D--------------A------

_____PET_____________________________________________________________

Homo 1 SDGIPMYKRNVMILTNPVAAKKNVSINTVTYEWAPPVQNQALARQYMQMLPKEKQPVAGSEGAQYRKKQLAKQLPAHDQD

Homo 2 --------------------------------------------------------------------------------

Chimp --------------------------------------------------------------------------------

Gorilla --------------------------------------------------------------------------------

Orangutan --------------------------------------------------------------------------------

Sheep --------------------------------------------------------------------------------

Cattle --------------------------------------------------------------------------------

Mouse 1 --------------------------------------------------------------------------------

Mouse 2 --------------------------------------------------------------------------------

Rat --------------------------------------------------------------------------------

Xenopus T----T---------S------D---------------------R--ELI--D---------------------------

LIM1

Homo 1 PSKCHELSPREVKEMEQFVKKYKSEALGVGDVKLPCEMDAQGPKQMNIPGGDRSTPAAVGAMEDKSAEHKRTQYSCYCCK

Homo 2 --------------------------------------------------------------------------------

Chimp --------------------------------------------------------------------------------

Gorilla --------------------------------------------------------V-----------------------

Orangutan --------------------------------------------------------------------------------

Sheep ---------K-------------------------RD-NT---NK-Y--------TT-----------------------

Cattle ---------K-------------N-----------RD-NT---NK-Y--------TT-----------------------

Mouse 1 ---------K-----------------------F-S--N---D KVHN-A-N-HA---- -SK-----S-K---------

Mouse 2 ---------K-----------------------F-S--N---D KVHN-A-N-HA---- -SK-----S-K---------

Rat ---------K-------------------------S--N---D KVHN-A---N------ SK-----A-K---------

Xenopus ---------N---Q---------N-V---------K-VE--ASGAGRSTN-SL--LTT-KSTD--V-AQ-GST-Y-FR--

LIM2______________

Homo 1 LSMKEGDPAIYAERAGYDKLWHPACFVCSTCHELLVDMIYFWKNEKLYCGRHYCDSEKPRCAGCDELIFSNEYTQAENQN

Homo 2 --------------------------------------------------------------------------------

Chimp --------------------------------------------------------------------------------

Gorilla --------------------------------------------------------------------------------

Orangutan --------------------------------------------------------------------------------

Sheep --------------------------------------------G-----------------------------------

Cattle --------------------------------------------G-----------------------------------

Mouse 1 HT-N--E-------------------I----G------------G-----------------------------------

Mouse 2 HT-N--E-------------------I----G------------G-----------------------------------

Rat NT-R----------------------I----G------------G-----------------------------------

Xenopus EN-R-----V-------------S----F--N------------G--------------------------------GL-

_ LIM3__________________________________

Homo 1 WHLKHFCCFDCDSILAGEIYVMVNDKPVCKPCYVKNHAVVCQGCHNAIDPEVQRVTYNNFSWHASTECFLCSCCSKCLIG

Homo 2 --------------------------------------------------------------------------------

Chimp --------------------------------------------------------------------------------

Gorilla --------------------------------------------------------------------------------

Orangutan --------------------------------------------------------------------------------

Sheep ------------N-------------------------------------------------------------------

Cattle ------------N-------------------------E-----------------------------------------

Mouse 1 ------------H----K-----T--------------------------------------------------------

Mouse 2 ------------H----K-----T--------------------------------------------------------

Rat ------------N----K-----R--------------------------------------------------------

Xenopus ------------IV------------A------------S---------------S--G-H---AP---I----------

Homo 1 QKFMPVEGMVFCSVECKKRMS

Homo 2 ---------------------

Chimp ---------------------

Gorilla ---------------------

Orangutan --------------------- LIM domain motif: CX_2_CX_16-23_HX_2_CX_2_CX_2_CX_16-21_CX_2-3_

Sheep ------------------M--

Cattle ------------------M--

Mouse 1 -----------------RM--

Mouse 2 -----------------RM--

Rat ------------------M--

Xenopus -----I------------K--S
